# Tapered Fiber-Optic Mach-Zehnder Interferometer for Ultra-High Sensitivity Measurement of Refractive Index

**DOI:** 10.3390/s19071652

**Published:** 2019-04-06

**Authors:** Vahid Ahsani, Farid Ahmed, Martin B.G. Jun, Colin Bradley

**Affiliations:** 1 Department of Mechanical Engineering, University of Victoria, Victoria, BC V8W 2Y2, Canada; mbgjun@purdue.edu (M.B.G.J.); cbr@uvic.ca (C.B.); 2 Department of Mechanical and Mechatronics Engineering, University of Waterloo, Waterloo, ON N2L 3G1, Canada; farid.ahmed@uwaterloo.ca; 3 School of Mechanical Engineering, Purdue University, West Lafayette, IN 47907, USA

**Keywords:** Mach-Zehnder interferometer, fiber-optic sensor, refractive index measurement, fiber tapering

## Abstract

A Mach-Zehnder interferometer (MZI) based fiberoptic refractive index (RI) sensor is constructed by uniformly tapering standard single mode fiber (SMF) for RI measurement. A custom flame-based tapering machine is used to fabricate microfiber MZI sensors directly from SMFs. The fabricated MZI device does not require any splicing of fibers and shows excellent RI sensitivity. The sensor with a cladding diameter of 35.5 µm and length of 20 mm exhibits RI sensitivity of 415 nm/RIU for RI range of 1.332 to 1.384, 1103 nm/RIU for RI range of 1.384 to 1.4204 and 4234 nm/RIU for RI range of 1.4204 to 1.4408, respectively. The sensor reveals a temperature sensitivity of 0.0097 nm/°C, which is relatively low in comparison to its ultra-high RI sensitivity. The proposed inexpensive and highly sensitive optical fiber RI sensors have numerous applications in chemical and biochemical sensing fields.

## 1. Introduction

Fiber optic refractive index (RI) sensors have attracted considerable attention for chemical and biochemical monitoring applications over the past few years [1,2] because of their useful characteristics, such as small size, high-resolution detection, excellent aging characteristics, ability to operate in chemically hazardous environments, and immunity to electromagnetic noise. Many researchers have tried to enhance the effectiveness of optical fiber RI sensors by improving sensitivity [3], enhancing resolution [4], simplifying fabrication techniques [5], dropping cost [6], increasing the robustness of sensor structure [7], and reducing insertion loss [8].

Gratings and interferometers are the two main configurations studied for fiber optic RI sensing [9]. Although long period gratings (LPGs) are a broadly used RI sensor [10,11,12,13,14], writing gratings are usually expensive and function only in narrow wavelength bands due to fiber gratings phase matching phenomenon. In-fiber interferometers such as Fabry-Perot interferometer (FPI), Michelson interferometer (MI), and Mach-Zehnder interferometer (MZI) have been introduced as alternative and viable approaches for RI sensing [15]. Also, the combination of interferometers and gratings has been reported in the literature; for instance, MZI has been constructed based on a pair of LPGs to further increase RI sensitivity [14,15].

Fiber-optic MZI sensors have been used in diverse monitoring applications, including ambient RI [16,17], temperature [18], pressure [19], torsion [20], and structural health [21] measurements. Recently, fiber MZI based RI sensing has gained considerable attention due to its enhanced sensitivity and fabrication simplicity. Alternative configurations for MZI sensors can be achieved utilizing various fiber types (such as multimode [6,15,22], microfiber [23], or photonic crystal fiber (PCF) [15,17,24] or fabrication techniques (such as surface plasmon resonance (SPR) [4,25,26], core mismatch [27,28,29], and tapering [15,30]). For example, concatenation of core-offset section and SMF abrupt taper is suggested to form an MZI [31]. This sensor revealed a relatively low RI sensitivity of 28.2 nm/RIU for a sensor length of 30 mm and its fabrication involved complex steps. In 2015, Zhao et al. [32] reported a 30 mm long all-fiber MZI-based RI sensor by splicing an SMF stub between two SMFs with small core offset at two splicing points. The fabricated sensor showed RI sensitivity of 78.7 nm/RIU in the range of 1.333 to 1.374 [32]. Although the fabrication process was simple and cost-effective, the reported RI sensitivity was relatively low. Another MZI sensor for RI measurement based on sandwiching and core-mismatched splicing of an SMF between two short sections of thin-core fibers was proposed by Rong et al. [27]. The maximum RI sensitivity of 159 nm/RIU for water-based solutions with an RI close to 1.33 was reported. In 2015, a PCF taper-based MZI for sensing changes in refractive index was presented with an RI sensitivity of 51.902 nm/RIU by Wu et al. [30]. The MZI sensor was fabricated by splicing a stub of PCF between two SMFs followed by PCF tapering. Such MZI configurations may not be feasible for many monitoring applications due to their weak mechanical strength and the use of expensive fiber. An inexpensive and simple-to-fabricate RI sensor with RI sensitivity of 158.4 nm/RIU based on two cascaded SMF tapers was demonstrated by Wang et al. in 2016 [33]. The fabrication of an MZI sensor for RI measurement from a long tapered single mode fiber was proposed by Yadav et al. [34]. The protein sensing device exhibited an RI sensitivity of about 1500 nm/RIU in the limited RI range of 1.3325 to 1.3377.

In this work, we present an ultra-high sensitivity, easy to fabricate, inexpensive, and mechanically robust in-line MZI based RI sensor constructed by tapering an SMF. A customized flame-based tapering machine was used to achieve sharp taper transitions and a uniform long taper waist in an SMF to create the MZI structure. For a specific taper waist length, the dependence of the sensor’s RI sensitivity on taper waist diameter (TWD) was investigated. In the RI range of 1.333 to 1.38, the RI sensitivities of 203 nm/RIU, 230 nm/RIU, 250 nm/RIU, 292 nm/RIU, and 415 nm/RIU were achieved for sensors with TWDs of 62 µm, 51.5 µm, 49 µm, 40 µm, and 35.5, respectively. A maximum RI sensitivity of 4234 nm/RIU was attained in the RI range of 1.4204 to 1.4408 for TWD 35.5 µm and taper waist length of 19.8 mm.

## 2. Principle of Sensor Operation

**Figure 1:**
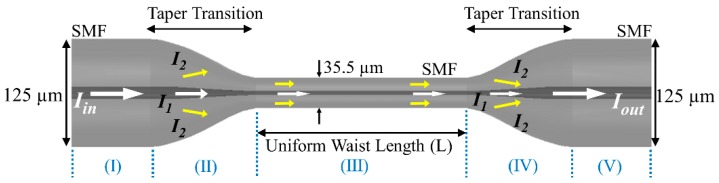
Schematic diagram of the internal structure of a microfiber MZI that was fabricated by employing the long uniform tapering technique.

Adiabatic tapering (both in down-taper and up-taper region) of SMF was used to achieve the MZI configuration. Such a down-taper region is shown to excite at least a few leaky modes, which then recombine with the core more at the up-taper region to produce an interference pattern [35]. Figure 1 provides a schematic representation of the long uniform tapered based Mach-Zehnder interferometer, which was constructed using an SMF. In the schematic, when light travels from region (I) to (II), higher order modes are excited, which travel along with the fundamental mode through the tapered region (III). Because of the significant difference between glass and air indices, the fundamental and higher order modes couple back together in the region (IV) to form an interferometric pattern. The resultant interference spectrum is described by the following formula [36]:

(1) $$I_{out}=I_{1}+I_{2}+2\sqrt{I_{1}I_{2}}\cos(\Delta \varphi )$$

where *I_out_*, *I*_1_, and *I*_2_ are the intensities of the interference signal, core, and cladding modes, respectively. Δφ is the phase difference between the core and cladding modes, which can be described by the following equation:

(2) $$\Delta \varphi =\frac{2\pi }{\lambda }(\Delta n_{eff})L$$

where λ is the light source central wavelength and *L* is the fiber uniform waist length. $\Delta n_{eff}$ is the variance between the effective RI of the core and cladding modes:

(3) $$\Delta n_{eff}=n_{eff}^{core}-n_{eff}^{cladding}$$

where $n_{eff}^{core}$ and $n_{eff}^{cladding}$ are the effective refractive indices of the core and cladding modes of the SMF, respectively. From Equations (1) and (2), it can be found that maximum transmission can happen when $\Delta \varphi =2\pi (\Delta n_{eff})L/\lambda =2m\pi$ (m is an integer). Therefore, the transmission signal shows peaks at the following wavelengths:

(4) $$\lambda_{m}=(\Delta n_{eff})L/m$$

$n_{eff}^{cladding}$ and $\Delta n_{eff}$ will change if the RI of the solution being measured is differed. $\Delta \lambda_{m}$ describes the m order shift of the interference spectrum and is given by:

(5) $$\Delta \lambda_{m}=\frac{(\Delta n_{eff}+\Delta n)L}{m}-\frac{\Delta n_{eff}L}{m}=\frac{\Delta nL}{m}$$

where Δn is the change in the RI of the measurand solution. Thus, from Equation (5), it can be seen that the variation of the transmission signal is a function of Δn when the length of the sensor (*L*) is constant.

## 3. Sensor Fabrication

**Figure 2:**
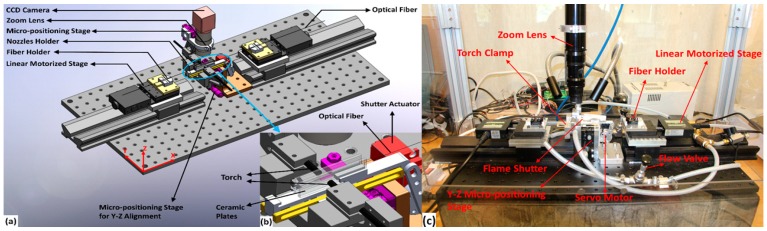
(**a**) Assembly model of the custom flame-based tapering machine and, (**b**) design of the sliding shutter mechanism to control heat delivery to the fiber, and (**c**) assembled custom flame- based tapering machine.

**Figure 3:**
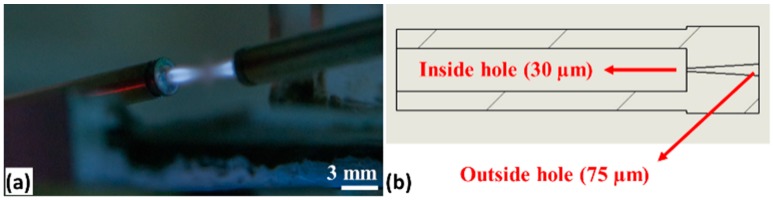
(**a**) Size of hydrogen flame used for long uniform tapering and, (**b**) the cross-section of the fabricated converging/diverging micro nozzle.

Figure 2a displays the Computer Aided Design (CAD) assembly model of the customized flame-based tapering machine designed for sensor fabrication. The enlarged image in Figure 2b shows the shutter mechanism integrated into the system to provide a controlled heat deposition into the fiber, which in turn offers an accurate geometry of the tapered profile. A pair of converging/diverging nozzles, as shown in Figure 3, was used to generate a heated volume that had a length of about 0.8 mm along the direction of the fiber axis. Hydrogen (99.99% pure) at a pressure of 20 psi was fed into the nozzles to avoid pollution on the tapered fiber. The flame temperature was controlled to remain above the fiber’s softening point, whilst keeping it below the glass’s melting point. For standard single mode fiber (SMF), the required temperature of 900 °C was maintained. After stripping and perfectly wiping the mid-section of an SMF with acetone, the fiber was clamped on two linear motorized stages. Travel distance, speed of each stage, and delay time to open or close the shutter were independently set in the tapering control software. The tapering parameters were tuned and optimized to obtain several tapered waist diameters, as shown in Table 1.

**Figure 4:**
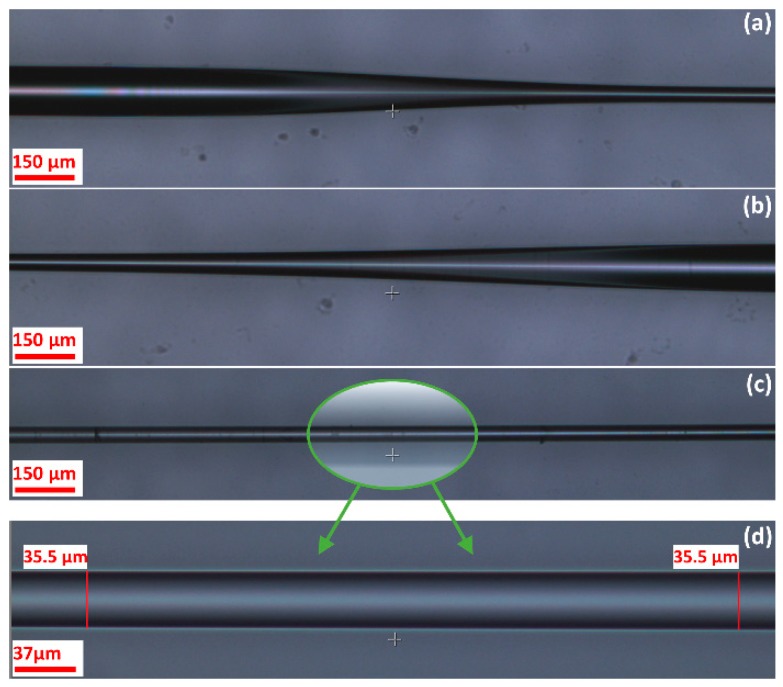
(**a**,**b**) SMF taper transition, (**c**) long uniform taper waist, (**d**) magnified image of the uniform taper waist.

The difference in the pulling and pushing speeds causes the fiber material to move in front of the flame and thus creates a long uniform taper profile. The generated sharp tapering angle split the core light into core and cladding light at the first transition region, while the second transition re-combined these two beams of light into a core light. Therefore, because of the optical path difference (OPD) between core and cladding arms, an interference fringe was generated. Although the core mode is restricted in the core, the transmission properties of the cladding modes can change as a result of the RI variation at the cladding-ambient interface. Smaller fiber diameter enables the cladding modes to reach closer to the measurand solution. Therefore, this enabled fabrication of sensors with improved RI sensitivity. Figure 4 shows the left and right taper transition profiles and uniform taper waist of 35.5 µm in the middle.

## 4. Results and Discussion

**Figure 5:**
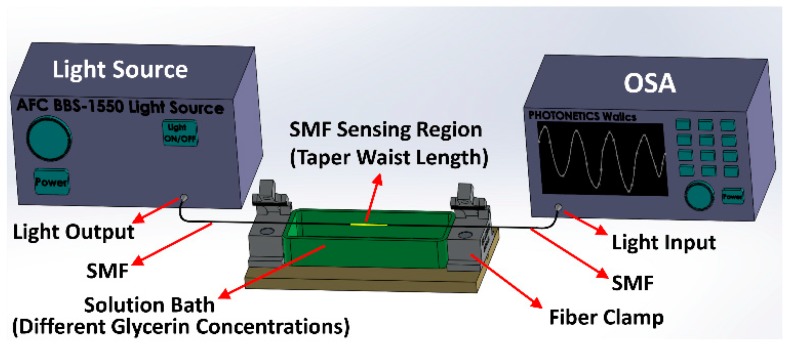
Schematic diagram of the experimental setup for refractive index characterization, OSA (Optical Spectrum Analyzer).

**Figure 6:**
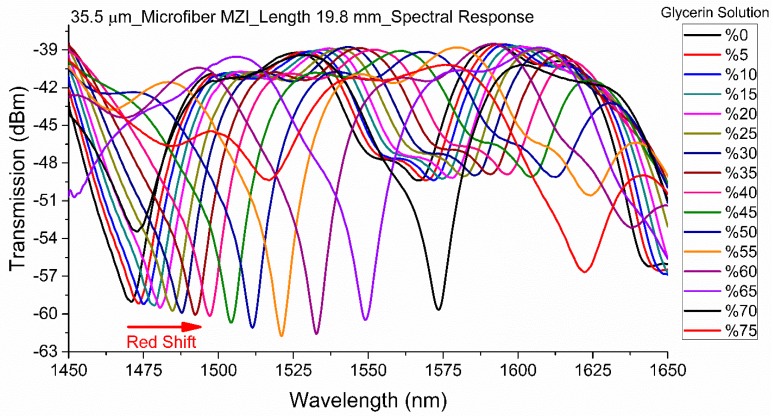
Spectral response of the MZI sensor with a 35.5 µm TWD to various concentrations of glycerin solution.

Due to the high sensitivity of MZI sensors to fiber twisting, the sensor was thoroughly secured on two fiber clamps in the RI characterization setup. Subsequently, the fiber was connected to a broadband light source (AMONICS ASLD-CWDM-5-B-FA, spectrum range: 1250–1650 nm) and a spectrum analyzer (PHOTONETICS Walics, Resolution: 0.02 nm, Spectrum range: 1450–1650 nm), to study the optical characteristics of the tapered microfiber MZI. The schematic representation of the RI characterization setup is illustrated in Figure 5. The response of interference fringes due to ambient RI change was examined for the MZIs with fiber TWDs of 62 µm, 51.5 µm, 49 µm, 40 µm, and 35.5 µm. These sensors were characterized with glycerin solutions of various concentrations at a room temperature of 22 °C. They were submerged in glycerin solutions of different RI ranging from 1.332 to 1.440 to achieve characterization data. After each step, the sensors were thoroughly cleaned with acetone, before immersion in an increasingly greater concentration of glycerin solution. Figure 6 illustrates the ambient RI dependent spectral response of the MZI sensor with TWD of 35.5 µm. The interference between broadband core mode and narrowband cladding mode may give rise to the asymmetric spectrum, as explained by the Fano interference phenomenon [37]. The MZI structure that generates such transmission interference is also very sensitive to an external perturbation such as ambient RI change. This might explain why this sensor is ultra-sensitive to RI.

**Figure 7:**
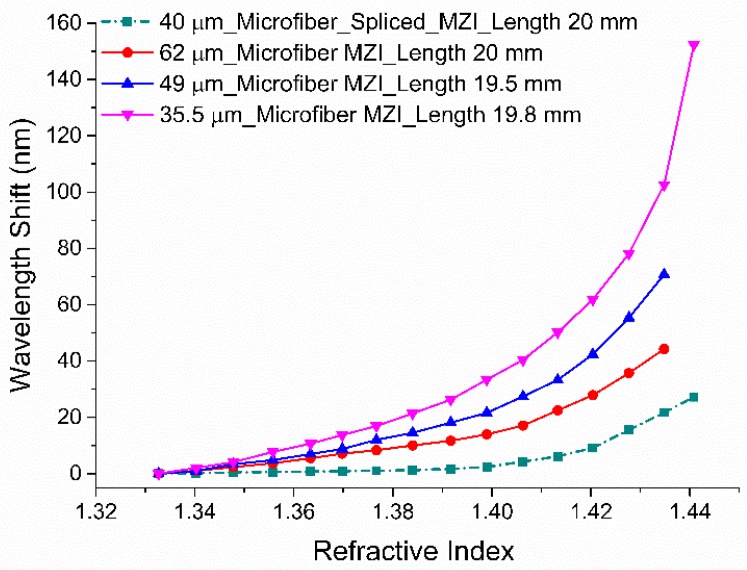
The spectral shift of the microfiber MZIs, with various waist diameters, due to changes in RI.

**Figure 8:**
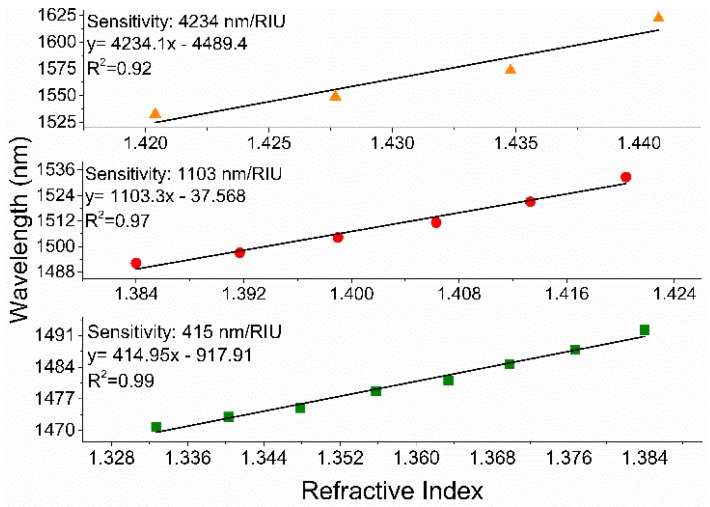
Linearization of the MZI sensor’s wavelength shift necessary to characterize sensitivity in three RI ranges. The characterized sensor has a TWD of 35.5 µm and taper waist length of 19.8 mm. The maximum RI sensitivity of ~ 4234 nm/RIU in the RI range of 1.4204 to 1.4408 was achieved.

Figure 7 displays the RI dependent wavelength shifts of three tapered MZIs with different TWDs. The plot also includes the absolute value of wavelength shifts due to ambient RI change of the RI sensor fabricated by splicing a microfiber (diameter: 40 µm) between a lead-in and lead-out SMFs. As the surrounding RI increases, a red-shift was observed in the MZI interference fringe. The performance of the microfiber MZIs were evaluated by their sensitivities, which were interpreted as the ratio of resonance wavelength shift to the variation in solution refractive index. The microfiber MZI with smallest TWD (35.5 µm) was chosen and its elaborated sensitivity graphs plotted in Figure 8; this shows more details of RI sensitivity analysis. Three subplots were generated from Figure 6 for index ranges of 1.3327 to 1.3840, 1.3840 to 1.4204 and 1.4204 to 1.4408 (Figure 8).

**Figure 9:**
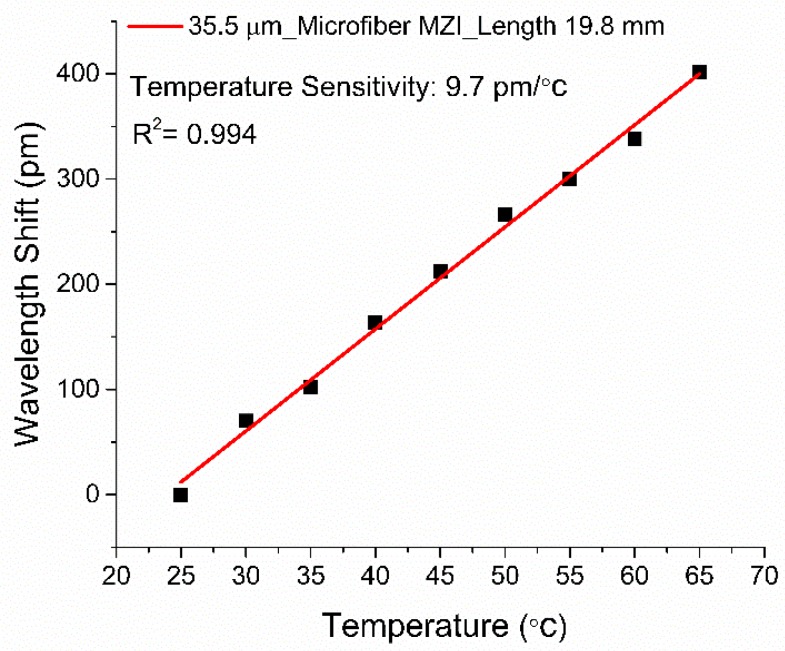
Temperature characterization of the microfiber MZI RI sensor with a TWD of 35.5 µm.

The effect of temperature cross-sensitivity needs to be considered in ambient RI measurement as the RI of most solutions changes with temperature variation. To determine the temperature response of the sensor configuration, an MZI sensor with a TWD of 35.5 µm and a length of 19.8 mm was placed in an oven for temperature characterization. The oven temperature gradually elevated from 25 °C to 65 °C in 5 °C increments. The correlation between the wavelength shift and the sensor’s temperature is shown in Figure 9. The temperature sensitivity of the MZI sensor is 0.0097 nm/°C. As a result, the RI measurement error, because of temperature effect, is approximately 2.33 × 10^−5^ RIU/°C and, therefore, negligible.

**Figure 10:**
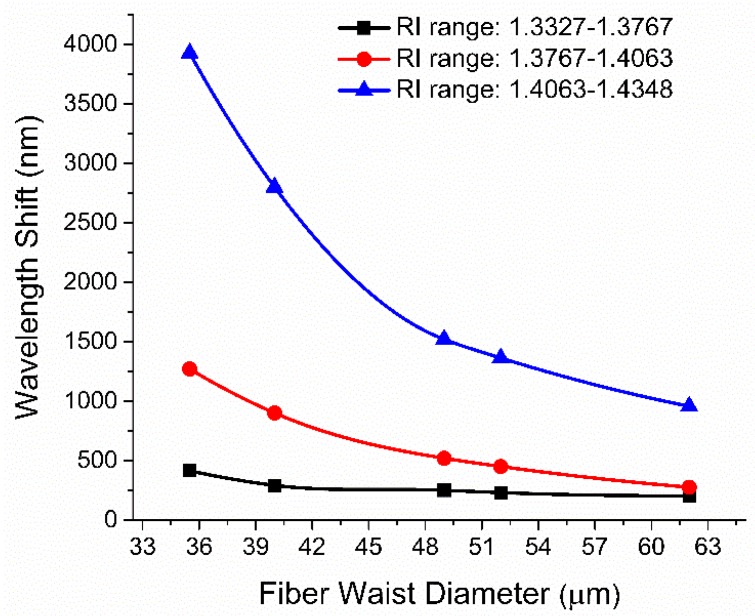
The relation between spectral wavelength shift and fiber waist diameter for various microfiber MZIs with different TWDS.

Figure 10 illustrates the effect of decreasing TWD (while keeping the taper waist length approximately constant) on RI sensitivity of the fabricated MZIs in various RI ranges. As the TWD decreases, the RI sensitivity of the fabricated MZIs increases nonlinearly. Microfiber MZIs with fiber diameters of 35.5 µm, 40 µm, 49 µm, 51.5 µm, and 62 µm were characterized for RI range from 1.3327 to 1.4348.

The results of the RI sensitivity analysis for microfiber MZIs with TWDs of 62 µm, 49 µm, and 35.5 µm, over six different refractive index ranges, are summarized in Table 2.

## 5. Conclusions

An easy and cost-effective fabrication of a fiber-optic MZI for ultra-high sensitivity RI measurement is proposed in this study. The MZI is constructed by tapering a standard SMF-28 fiber. Several MZIs, with constant taper length but different uniform TWDs, were fabricated to investigate the sensor’s structural influence on RI measurement. The MZIs with uniform TWDs of 62 µm, 49 µm, and 35.5 µm show the maximum sensitivities of 956 nm/RIU, 1520 nm/RIU, and 4234 nm/RIU, respectively. The MZI device shows a temperature sensitivity of 0.0097 nm/°C, which is insignificant relative to its RI sensitivity. Since the fabrication of this sensor does not require any splicing, it is likely to have insignificant insertion losses and stronger mechanical properties compared to spliced microfiber MZI. Furthermore, the high sensitivity characteristic of Mach-Zehnder interferometers makes them suitable for chemical, biochemical, and biological sensing in an aqueous environment.

## Figures and Tables

**Table 1:** The manufacturing process parameters controlled to fabricate the sensors with a range of sensitivities.

Pushing Speed (µm/s)	Pushing Distance (mm)	Pulling Speed (µm/s)	Pulling Distance (mm)	Shutter Open Delay (ms)	Shutter Close Delay (ms)	TWD (µm)
25	5	100	20	2000	0	62
25	3.5	140	19.6	2000	500	51.5
30	3	195	19.5	2000	500	49
25	2.2	225	19.6	2000	500	40
25	1.8	275	19.8	2000	500	35.5

**Table 2:** The RI sensitivity of three microfiber MZIs with various TWDs and constant taper lengths are shown for different RI ranges.

	RI Range	1.3327 to 1.3767	1.3767 to 1.4063	1.4063 to 1.4348	1.3327 to 1.3840	1.3840 to 1.4204	1.4204 to 1.4408
TWD							
Microfiber MZI (62 µm)		203 nm/RIU	290 nm/RIU	957 nm/RIU	NA	NA	NA
Microfiber MZI (49 µm)		277 nm/RIU	550 nm/RIU	1520 nm/RIU	NA	NA	NA
Microfiber MZI (35.5 µm)		NA	NA	NA	415 nm/RIU	1103 nm/RIU	4234 nm/RIU
